# Birds-YOLO: A Bird Detection Model for Dongting Lake Based on Modified YOLOv11

**DOI:** 10.3390/biology14111515

**Published:** 2025-10-29

**Authors:** Shuai Fang, Yue Shen, Haojie Zou, Yerong Yin, Wei Jin, Haoyu Zhou

**Affiliations:** College of Information and Intelligent Science and Technology, Hunan Agricultural University, Changsha 410128, China; fs@stu.hunau.edu.cn (S.F.); shenyue@hunau.edu.cn (Y.S.); zouhaojie@stu.hunau.edu.cn (H.Z.); yyrsz0796@stu.hunau.edu.cn (Y.Y.); jweiwei77@stu.hunau.edu.cn (W.J.)

**Keywords:** bird detection, Dongting Lake, YOLOv11, attention mechanism, feature fusion

## Abstract

**Simple Summary:**

Detecting birds in natural environments like Dongting Lake is challenging due to cluttered backgrounds, birds of different sizes, and a wide variety of species. This study aimed to create a more accurate and efficient model to detect birds in such complex settings. We collected real-world images of 47 bird species from Dongting Lake and developed an improved detection system that better recognizes birds in various conditions. Our model improves the way it focuses on important details and enhances the ability to identify birds of different sizes. Tests show that our model performs significantly better than the basic version, with higher success rates in detecting birds correctly while missing fewer birds. It works well on both public bird image collections and our own field data. This advancement helps create more reliable tools for monitoring bird populations in the wild, which is important for protecting biodiversity and understanding ecosystem health. The improved model can support conservation efforts by enabling automated, real-time bird observation in natural habitats, making ecological research more efficient and scalable.

**Abstract:**

To address the challenges posed by complex background interference, varying target sizes, and high species diversity in bird detection tasks in the Dongting Lake region, this paper proposes an enhanced bird detection model named Birds-YOLO, based on the YOLOv11 framework. First, the EMA mechanism is introduced to replace the original C2PSA module. This mechanism synchronously captures global dependencies in the channel dimension and local detailed features in the spatial dimension, thereby enhancing the model’s robustness in cluttered environments. Second, the model incorporates an improved RepNCSPELAN4-ECO module, by reasonably integrating depthwise separable convolution modules and combining them with an adaptive channel compression mechanism, to strengthen feature extraction and multi-scale feature fusion, effectively enhances the detection capability for bird targets at different scales. Finally, the neck component of the network is redesigned using lightweight GSConv convolution, which integrates the principles of grouped and spatial convolutions. This design preserves the feature modeling capacity of standard convolution while incorporating the computational efficiency of depthwise separable convolution, thereby reducing model complexity without sacrificing accuracy. Experimental results show that, compared to the baseline YOLOv11n, Birds-YOLO achieves a 5.0% improvement in recall and a 3.5% increase in mAP@0.5 on the CUB200-2011 dataset. On the in-house DTH-Birds dataset, it gains 3.7% in precision, 3.7% in recall, and 2.6% in mAP@0.5, demonstrating consistent performance enhancement across both public and private benchmarks. The model’s generalization ability and robustness are further validated through extensive ablation studies and comparative experiments, indicating its strong potential for practical deployment in bird detection tasks in complex natural environments such as Dongting Lake.

## 1. Introduction

As a critical transit hub for the habitat and migration of numerous bird species, Dongting Lake supports a rich wetland ecosystem that offers a unique and favorable environment for avian life. This makes it an ideal natural site for investigating bird species diversity. Changes in avian diversity serve as a key ecological indicator, reflecting shifts in the environmental conditions of the Dongting Lake wetland, and thus hold significant value for both scientific research and ecological monitoring. Within this context, the task of automated bird detection functions as an “intelligent sentinel” in biodiversity surveys [1,2]. It enables the efficient and accurate identification and localization of bird targets, substantially enhancing both the efficiency of field surveys and the reliability of collected ecological data [3].

Traditional bird detection methods rely primarily on manual inspection of images or video frames by domain experts for species identification and data recording. However, this approach is labor-intensive, costly, and inefficient when applied to large-scale datasets. Moreover, detection results often lack consistency due to inter-observer variability in expertise and recognition ability. These limitations are further aggravated in complex environments, where visual clutter significantly degrades detection accuracy.

In contrast to manual detection, computer vision-based bird detection leverages efficient algorithms to rapidly process large-scale datasets, thereby significantly enhancing data processing efficiency and enabling precise extraction of bird-related features. Current mainstream detection approaches can be broadly categorized into three groups: one-stage detection methods, two-stage detection methods, and Transformer-based end-to-end detection methods. Two-stage detection methods, exemplified by the widely used Faster R-CNN [4,5,6], first employ a Region Proposal Network (RPN) to generate candidate object regions, which are subsequently classified and refined using bounding box regression. While such methods typically achieve high detection accuracy, their multi-stage processing pipelines lead to relatively slow inference speeds, making them less suitable for real-time applications. Transformer-based detection models, such as the representative DETR [7,8], exhibit strong global feature modeling capabilities and effectively capture long-range dependencies within images. However, these models are often characterized by large parameter sizes, slow convergence during training, and a high demand for computational resources. These limitations pose challenges for deployment in resource-constrained environments or scenarios requiring real-time performance.

In contrast, one-stage detection methods such as YOLO (You Only Look Once) [9,10,11,12,13] and RetinaNet [14], achieve significantly higher detection speeds by eliminating the region proposal stage and directly predicting object categories and locations on the feature maps. Among these, the YOLO family of models demonstrates a favorable balance between real-time processing capability, detection accuracy, and generalization performance, making it particularly well-suited for large-scale, long-duration monitoring tasks. Given these advantages, this study adopts YOLOv11 [15] as the baseline model to investigate bird detection in the Dongting Lake region.

Although YOLOv11, as the latest iteration of the YOLO series, has made significant progress in algorithm architecture and performance optimization, it still faces multiple challenges in the bird detection task in Dongting Lake. For example, the recognition of small-scale bird targets is still difficult, the uneven distribution of target scales restricts the generalization ability of the model, and there is also noise interference caused by similar backgrounds. To address the challenge of large-scale variations in bird images, researchers have conducted extensive investigations from the perspectives of network architecture optimization and learning strategy innovation. In object detection tasks, a common approach is to employ feature pyramid structures [16] to integrate multi-resolution feature maps, thereby facilitating multi-scale representation. Additionally, some methods incorporate multi-scale modules within single-branch networks. For instance, the MSCB module in MSCAM [17] draws inspiration from the Inception architecture, utilizing parallel convolutions with kernel sizes of 1 × 1, 3 × 3, and 5 × 5 to capture features at different scales and enhance the network’s representational capacity. The Atrous Spatial Pyramid Pooling (ASPP) [18] module improves contextual awareness through a dilated convolution pyramid. However, these manually crafted convolutional configurations often perform poorly when adapting to diverse scale distributions in complex scenes. In recent years, multi-task learning in object detection has mainly concentrated on the joint optimization of classification and localization sub-tasks. Some methods have also introduced auxiliary tasks (such as semantic segmentation or keypoint detection) to enhance the model’s understanding of object structures and contextual information. Nevertheless, the effectiveness of auxiliary tasks largely depends on their relevance to the main task; poorly correlated auxiliary tasks may lead to gradient interference or overfitting, ultimately undermining the model’s generalization performance. Simplifying them will facilitate better understanding for a broader audience.

In the detection task of bird images, background interference is one of the key challenges affecting model performance. In natural scenes, bird targets are often highly fused with background elements such as leaves, branches, sky, water, etc., leading to blurred target boundaries and feature confusion, which in turn triggers false or missed detection [19]. To address this issue, researchers have delved into three avenues: feature enhancement, context modeling, and attention focusing. In terms of feature enhancement, early approaches aimed to amplify the texture disparity between the target and the background through the utilization of a background-suppressing convolution kernel or an edge-aware loss function. However, these methods exhibit limited efficacy when the background and the target share similar color distributions. In terms of context modeling, researchers often use GNN [20] or Transformer architectures to construct semantic relationship graphs between the target and the environment/other objects, such as human-object interaction graphs or multi-target tracking graphs, to assist in distinguishing between the target and the background in interaction detection and character association tasks. Although the approach improves context understanding, the construction of additional relationship graphs relies on a large number of annotations, which becomes an important bottleneck constraining the generalization ability. Meanwhile, the attention mechanism becomes a core tool for suppressing background interference, such as the MCA [21], which enables the model to focus on target saliency features by dynamically suppressing background-related channels and spatial regions; the CBAM [22] module further combines the spatial and channel attention to provide a more comprehensive and effective feature extraction capability.

The YOLO series detection methods are also widely used in small target detection and ecological monitoring. For example, Li et al. [23] proposed an improved YOLOv8 based SOD-YOLO, combined with RFCBAM and BSSI-FPN modules to optimize small-scale object detection and multi-scale feature fusion capabilities. Wang et al. [24] proposed a WB-YOLO integrated visual transformer encoder module based on improved YOLOv7, which is used for efficient detection of wild bats to achieve effective multi-scale feature fusio. Zhang et al. [25] propose YOLO-Feature and Clustering Enhanced (YOLO-FCE), an improved model based on the YOLOv9 architecture to evaluate and enhance the model’s feature extraction capabilities. Ji et al. [26] Propose HydroSpot-YOLO, which integrates an Attentional Scale Sequence Fusion (ASF) mechanism and a P2 detection layer to improve the detection of small and densely clustered targets under challenging conditions such as water reflections, cluttered backgrounds, and variable illumination. Although these methods based on Yolo have promoted the development of ecological monitoring, there are still some limitations. Some methods introduce complex modules, increase the computational cost, limit their deployment in the resource constrained field environment, or do not fully consider the unique challenges of ecological data, such as extreme scale changes, high background clutter, species similarity and limited annotation data sets. In addition, some models sacrifice reasoning efficiency to obtain accuracy or lack robustness in different ecological scenarios.

To address the aforementioned challenges, this study proposes targeted enhancements based on the YOLOv11n framework, aiming to improve target representation and scene adaptability within the complex environment of Dongting Lake. The main contributions of this work are summarized as follows:

1. The introduction of an Efficient Multiscale Attention Mechanism (EMA) [27] enhances the feature representation of targets across different scales by employing parallel subnetworks that simultaneously capture both channel-wise and spatial information. This mechanism significantly improves the model’s capability for multi-scale detection in complex background scenarios.
2. The improved RepNCSPELAN4-ECO module is designed to introduce depthwise separable convolution (DWConv) and adaptive channel compression mechanisms, making feature extraction and multi-scale feature fusion more comprehensive and efficient, and improving the detection capability of birds at different scales;
3. Neck network ordinary convolution is upgraded to lightweight GSConv [28] convolution, which effectively aggregates the global context and yet significantly reduces the computational redundancy through the combination of grouped convolution and spatial convolution, improving the accuracy and speed of detection.
4. We constructed the Dongting Lake bird detection dataset DTH-Birds by photographing high-quality common birds in Dongting Lake through various ways and performed data labeling and data enhancement, totaling 14,107 images. Meanwhile, the robustness of the Birds-YOLO model in dealing with diverse datasets and different scenarios are further demonstrated on the open bird dataset CUB200-2011 [29].

## 2. Materials and Methods

### 2.1. Data Acquisition Processing

**Figure 1:**
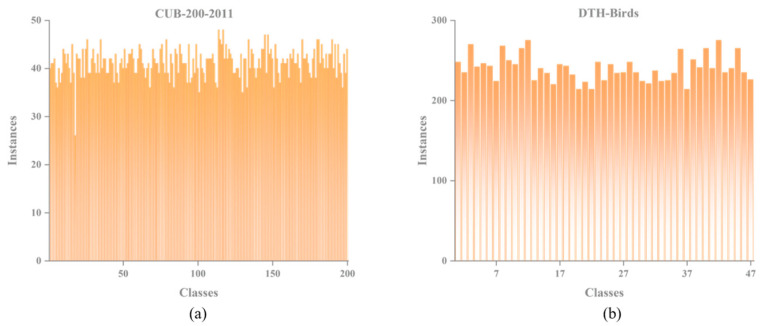
Comparison of bird species and training examples in CUB-200-2011 dataset and DTH-Birds dataset. (**a**) Number of bird species and training examples in the CUB-200-2011 dataset; (**b**) Number of bird species and training examples in the DTH-Birds dataset.

In this study, the improved Birds-YOLO model is evaluated on the publicly available CUB-200-2011 dataset and a self-constructed dataset, DTH-Birds, as illustrated in Figure 1. The CUB-200-2011 dataset is a widely used benchmark for bird image classification, comprising 11,788 images across 200 bird subcategories. Each image is annotated with a category label and bounding box coordinates indicating the bird’s location. The dataset is typically split into training, validation, and test sets in a 70%:15%:15% ratio, containing 8242, 1773, and 1773 images, respectively.

The DTH-Birds dataset was collected from early 2024 to March 2025. Data sources include the bird-watching monitoring platform provided by the Hunan Provincial Department of Natural Resources and field photographs taken by researchers at Dongting Lake. The dataset was assembled through two main collection phases: the first involved video recording, screenshot extraction, cropping, and organization of images from the monitoring platform between January and February 2024; the second consisted of on-site field photography conducted in the Dongting Lake area in March 2025. The collected images encompass a wide range of weather conditions, varying degrees of occlusion, differences in bird density, and scale variations. This diversity enhances the dataset’s representativeness and contributes to improving the model’s generalization capability.

**Figure 2:**
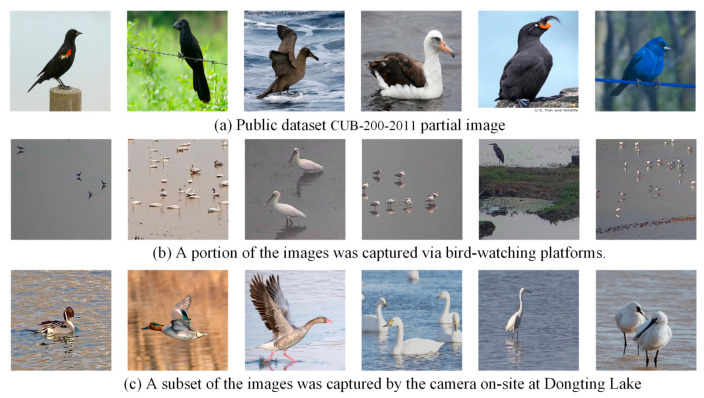
Dataset sample image. Sub-figure (**a**) shows the public dataset CUB-200-2011 partial image (**b**) shows a portion of the images captured via bird-watching platforms (**c**) shows a subset of` the images captured by the camera on-site at Dongting Lake.

The DTH-Birds dataset exhibits significant differences in diversity and background complexity compared to CUB-200-2011, as illustrated in Figure 2. Although CUB-200-2011 covers 200 bird subclasses and provides accurate bounding box annotations, its images are mostly captured by networks and manually filtered, with relatively clean backgrounds, concentrated shooting angles, stable lighting conditions, and relatively limited scene changes. The DTH-Birds dataset is directly collected from real ecological monitoring and field shooting environments in the Dongting Lake area of Hunan Province. It includes both long-distance monitoring images from bird watching platform video screenshots and high-resolution images taken up close in the field, covering various weather conditions such as sunny and cloudy days, as well as complex scenarios such as water reflection, vegetation obstruction, dense bird populations, and sparse individuals. This collection method makes DTH-Birds closer to real monitoring task scenarios in terms of background interference, target scale changes, viewpoint diversity, and environmental dynamics, which puts higher demands on the robustness and generalization ability of the model.

**Figure 3:**
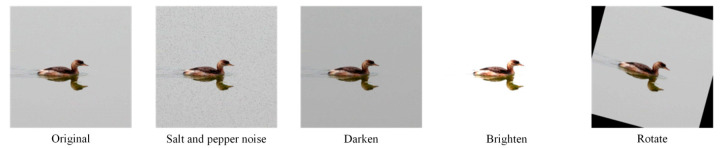
Data augmentation examples on the DTH-Birds dataset. Original images are augmented with salt-and-pepper noise, darkening, lightening, and rotation to increase diversity and robustness.

To address the challenges of limited availability and imbalanced sample numbers across certain bird categories, four data augmentation techniques were applied to selected classes (illustrated in Figure 3). These techniques include adding salt-and-pepper noise, darkening, lightening, and rotating, aimed at increasing image diversity and improving category distribution balance. The final dataset comprises 14,107 images spanning 47 bird subclasses. It is partitioned into training, validation, and test sets at an 80%:10%:10% ratio, containing 11,287, 1410, and 1410 images, respectively. The specific dataset partitioning and data augmentation parameters are shown in Table 1.

The data annotation process was performed using the LabelImg tool, followed by expert-guided screening, classification, and refinement. The annotation adhered to the following core principles: bounding boxes must accurately and fully enclose the entire body of the target bird; in images containing multiple bird species, each category is labeled separately; in densely populated scenes, individual birds are annotated and distinguished one by one; partially occluded birds are annotated based on their visible regions.

### 2.2. YOLOv11’s Network Architecture

**Figure 4:**
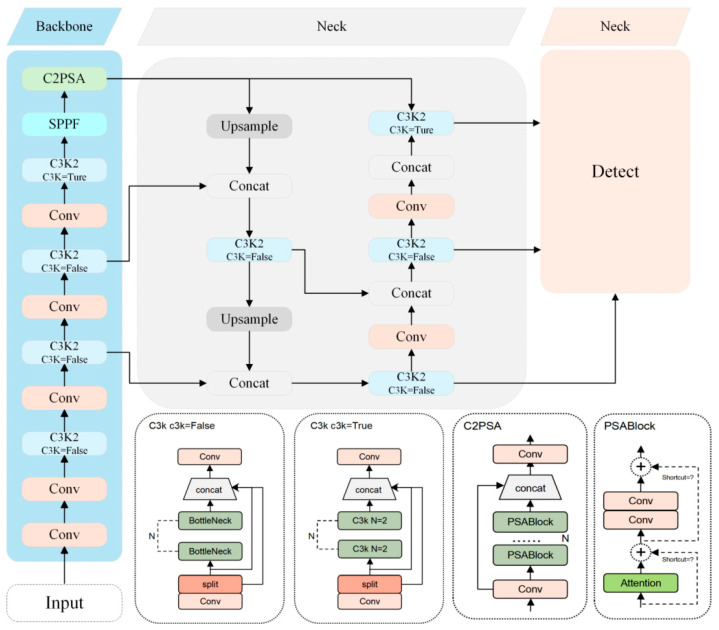
YOLOv11 Network Architecture Diagram.

YOLOv11 is a real-time object detection algorithm officially released by Ultralytics on 30 September 2024. Its core design goal is to balance detection accuracy and inference speed while systematically optimizing object detection performance in complex scenes. Compared to previous versions in the YOLO series, the YOLOv11 architecture (illustrated in Figure 4) primarily consists of three components: the backbone, neck, and head.

Regarding the backbone network design, YOLOv11 employs CSPDarknet53 as its feature extraction backbone and generates multi-scale feature maps through five subsampling stages to enhance the model’s ability to perceive targets at varying scales. Key modules include: The C3k2 module replaces the traditional C2f [30] structure, optimizing residual connections and inter-channel feature interactions, thereby significantly improving the model’s multi-scale feature representation capability. The CBS module, consisting of convolution, batch normalization, and the SiLU [31] activation function, enables fast nonlinear transformations and feature map normalization, which helps stabilize training and enhance representational power. The Spatial Pyramid Pooling Fast (SPPF) module applies multi-scale pooling operations to map feature maps to fixed dimensions, effectively strengthening the model’s global semantic information extraction. The C2PSA module integrates pyramid slice attention by combining multi-level feature slicing with channel-spatial attention fusion, improving the model’s discriminative ability in complex backgrounds and small-object scenarios [32].

In terms of neck network design, YOLOv11 adopts a PAN-FPN structure, which enhances the bidirectional flow of information through both bottom-up and top-down path aggregation. This design strengthens the integration of shallow spatial features and deep semantic features, effectively compensating for the limitations of the traditional FPN [33] in terms of localization accuracy. For the detection head, YOLOv11 employs a decoupled architecture that separately handles classification and bounding box regression tasks, thereby improving task-specific optimization. The classification branch utilizes Binary Cross-Entropy (BCE) loss and incorporates two layers of DWConv, which significantly reduces model complexity and computational cost while preserving strong classification performance. The regression branch combines Distribution Focal Loss (DFL) and Complete Intersection over Union (CIoU [34]) loss to jointly optimize bounding box localization accuracy and regression stability [16].

Overall, YOLOv11 enhances real-time object detection performance through lightweight optimization of the backbone network, improved multi-scale feature fusion mechanisms, an efficient decoupled detection head design, and targeted enhancements for small object detection. Based on these advantages, this study selects YOLOv11n as the baseline model for further improvement and experimentation [35].

### 2.3. Birds-YOLO’s Network Architecture

Although YOLOv11n demonstrates an excellent balance between real-time performance and detection accuracy in general object detection tasks, it still exhibits notable limitations in fine-grained feature representation, scale adaptability, and multi-level feature fusion efficiency when applied to multi-scale bird detection in the complex ecological environment of the Dongting Lake Basin. Specifically, the small object sizes, dense spatial distribution, and complex background interference of bird targets in this region pose significant challenges for YOLOv11n in terms of feature expressiveness and detection robustness. To address these issues, this paper proposes an enhanced architecture, Birds-YOLO, optimized specifically for bird detection. The proposed model improves feature extraction, multi-scale adaptability, and generalization performance through targeted structural modifications.

**Figure 5:**
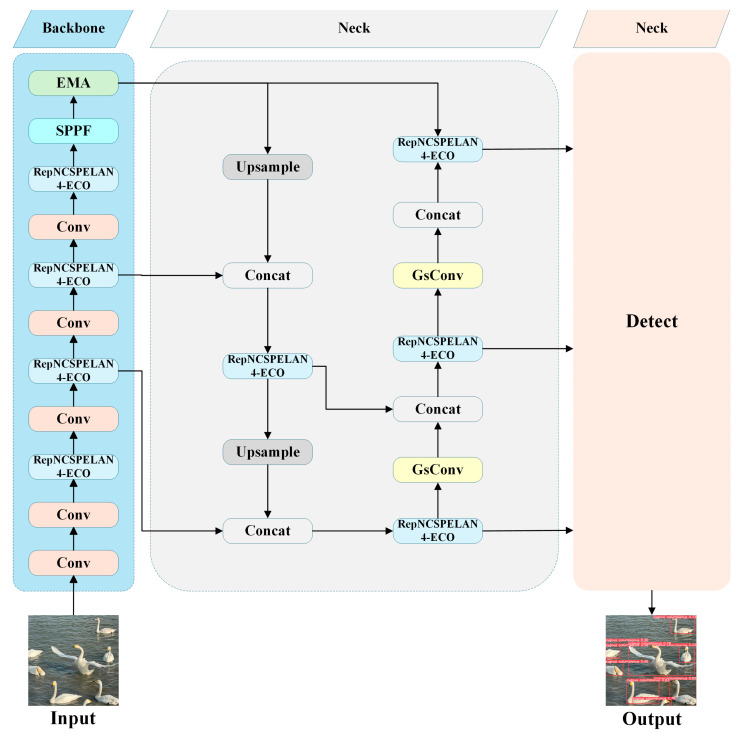
Birds-YOLO Network Architecture Diagram.

The network architecture of Birds-YOLO illustrated in Figure 5, retains the hierarchical structure of the classic YOLO series, comprising three key components: backbone, neck, and head. To address the core challenges associated with bird detection in the Dongting Lake region, the following architectural enhancements are introduced:

To tackle the issues of large-scale variation and complex background interference, EMA is incorporated to dynamically enhance feature representations across different scales. A multi-branch parallel processing strategy is employed to simultaneously capture channel-wise and spatial positional weights. Through feature fusion, a refined multi-scale attention map is generated, thereby improving the model’s ability to detect targets of varying scales in cluttered backgrounds.

To further optimize the efficiency of feature extraction and multi-scale fusion, the RepNCSPELAN4-ECO module is improved by introducing depthwise separable convolution and adaptive channel compression mechanism. These changes enhance the module’s ability to capture features of birds of different sizes. The introduction of dynamic channel compression mechanism, combined with residual connections and cross scale feature interaction, significantly improves the adequacy of feature representation and the efficiency of multi-scale feature integration.

To reduce the computational redundancy commonly found in traditional convolution operations within the neck network, standard convolutions are replaced with lightweight GSConv modules. GSConv integrates grouped convolution and spatial convolution in a collaborative design that reduces computational complexity while preserving global context aggregation. Specifically, it partitions the input feature map into multiple groups, applies spatial convolution independently within each group, and employs a channel shuffle mechanism to promote inter-group information exchange. This design improves inference speed without compromising detection accuracy.

Through the aforementioned enhancements, Birds-YOLO significantly improves the feature extraction capability and detection robustness for multi-scale bird targets in complex wetland environments while maintaining competitive overall detection performance.

### 2.4. EMA

**Figure 6:**
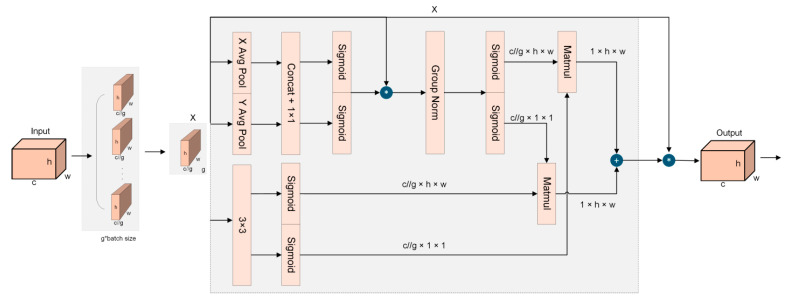
The structure of the EMA module.

In the Dongting Lake wetland ecological monitoring task, the target detection algorithm faces two core challenges: first, due to the variability of target distance, flight attitude, and habitat during the shooting process, the size span of birds in the image is significant, which makes it difficult for the traditional model to take into account the global semantic representation of large targets and fine-grained texture information of small targets in feature extraction; Secondly, the background of the basin is complex and dynamic, and there is high similarity interference between natural elements such as wetland vegetation, water reflection, reed bushes and bird targets. In addition, environmental factors such as light changes and seasonal vegetation coverage differences further aggravate the difficulty of distinguishing between targets and backgrounds, making the model prone to false detection or missing detection in complex scenes. In response to the above challenges, this study introduced an efficient multi-scale attention (EMA) mechanism into the yolov11n model to dynamically enhance the feature representation ability of targets with different scales and the inhibition ability of the model against complex backgrounds, so as to improve the detection robustness of the algorithm in the wetland environment. The EMA structure is shown in Figure 6.

The EMA module operates through an excitation mechanism and a modulation mechanism. The excitation mechanism computes the inner product between the input feature and a learnable parameter to generate a similarity matrix, where each element measures the semantic similarity between a feature position and the parameter. Higher similarity indicates greater feature importance under the current context. Subsequently, the modulation mechanism dynamically reweights each feature position based on this matrix, enhancing informative features while suppressing redundant ones.

The EMA module employs a multi-scale attention mechanism to capture channel and spatial information simultaneously through a parallel sub-network. It mainly consists of two branches: 1 × 1 branch and 3 × 3 branch. The channel and spatial information are effectively combined through these two parallel-designed branches without adding too many parameters and computational costs. An input feature map $X\in R^{C\times H\times W}$ is first divided into G sub-features in the channel direction, i.e., $X=[X_{0},X_{1},\ldots ,X_{G-1}]X\in R^{C\times H\times W}$. The divided G sub-feature maps are fused with other branch information on branch 1; Branch 2 uses two-dimensional average pooling to globally average the feature maps from both height and width directions, as shown in

(1) $$Z_{c}=\frac{1}{H\times W}\sum_{j}^{H}\sum_{i}^{W}X_{c}(i,j),$$

where H and W denote the height and width of the feature map; Xc denotes the feature tensor of different channels. Each sub-feature group Xi after 1 × 1 convolution results in $Y_{i}^{1\times 1}=\sigma (W_{1\times 1}\times X_{i}),Y_{i}^{1\times 1}\in R^{\frac{C}{G}\times H\times W}$, $Y_{i}^{3\times 3}=\sigma (W_{3\times 3}\times X_{i}),Y_{i}^{3\times 3}\in R^{\frac{C}{G}\times H\times W}$, W1 × 1 is the weight matrix of 1 × 1 convolution and W3 × 3 is the weight matrix of 3 × 3 convolution, which is the activation function. The outputs of the 1 × 1 branch and the 3 × 3 branch are fused by cross-dimensional interaction, and the outputs are aggregated for all sub-feature groups $Y_{i}=Y_{i}^{1\times 1}+Y_{i}^{3\times 3}$, $Y=[Y_{0},Y_{1},\ldots ,Y_{G-1}]$.

The EMA module enhances feature representation by integrating channel and spatial information through feature grouping and a multi-scale parallel sub-network. It adaptively weights feature maps according to their importance, improving multi-scale bird detection. By smoothing the attention distribution, EMA reduces sensitivity to noise and outliers, thereby increasing robustness. Moreover, its grouped and parallel design achieves strong feature expressiveness with low computational overhead.

### 2.5. RepNCSPELAN4-ECO

**Figure 7:**
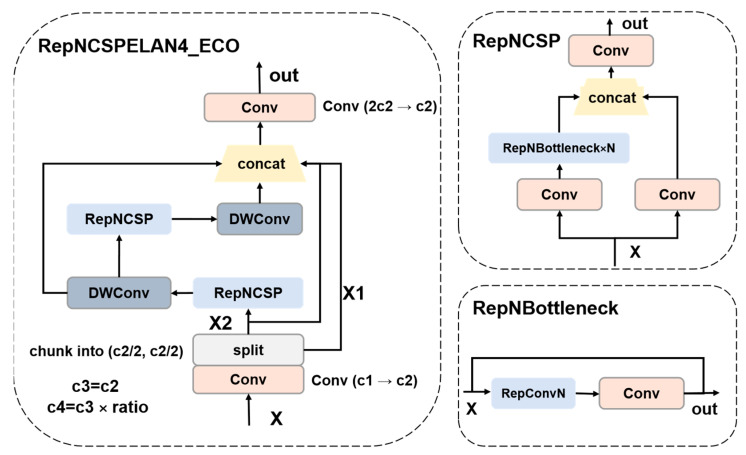
The structure of the RepNCSPELAN4-ECO module.

On the basis of the original RepNCSPELAN4 module [36], RepNCSPELAN4_ECO introduces multiple key structural optimizations aimed at achieving a better balance between high-precision feature extraction and efficient computation, particularly suitable for fine-grained object detection tasks in long-distance bird detection. The main improvements are reflected in the following aspects:

Using DWConv instead of standard convolution. RepNCSPELAN4 uses standard 3 × 3 convolution for spatial feature extraction in branch paths, which has strong expressive power but comes with high computational overhead. To address this issue, RepNCSPELAN4_ECO replaced both standard convolution operations in the branch with DWConv, located on the cv2 and cv3 paths, respectively. The computational complexity of standard convolution is O (H × W × C_in_ × C_out_ × K^2^), and K = 3 is the size of the convolution kernel; DWConv decomposes convolution into two steps: depthwise and Pointwise (1 × 1), reducing its complexity to O (H × W × C × K^2^ + H × W × C^2^), where C = C_in_ = C_out_. At channel number under the typical setting of C = 256, the computational cost of a single 3 × 3 convolution can be reduced by approximately C/K^2^ ≈ 28.4 times, significantly reducing the FLOPs and memory bandwidth requirements of the RepNCSPELAN4 model. The structural details of the RepNCSPELAN4-ECO module are illustrated in Figure 7.

The original RepNCSPELAN4 module requires manual specification of the intermediate channel dimensions c3 and c4, complicating parameter configuration and limiting scalability and automation. To address this, RepNCSPELAN4_ECO introduces a proportional control mechanism that automatically computes the intermediate channel count as c_4_ = int(c_2_ × ratio), where c_2_ is the output channel number and ratio is usually set to 0.5. This design simplifies network configuration, enables flexible model width scaling via the ratio, and enhances the module’s generality and scalability.

Despite its lightweight design, RepNCSPELAN4_ECO preserves the multi-path information aggregation mechanism of RepNCSPELAN4. the input is convolved by 1 × 1 and split into two parts through chunk (2, 1); Part of it is directly connected and retained (y[0]), while the other undergoes deep nonlinear transformation through two cascaded RepNCSP + DWConv modules. Finally, y[0] the output of the first stage, and the output of the second stage are concatenated along the channel dimension and fused by a 1 × 1 convolution. This design inherits the gradient flow optimization of ELAN and the feature reuse principle of CSP, ensuring effective integration of shallow details and deep semantics while minimizing redundancy and preserving feature expressiveness.

Overall, RepNCSPELAN4-ECO effectively reduces redundant computational overhead by introducing DWConv and adaptive channel compression mechanisms. The improved architecture achieves a better balance between accuracy and speed without significantly sacrificing feature expression capabilities, making it suitable for object detection tasks in resource constrained scenarios.

### 2.6. GSConv

GSConv effectively optimizes traditional convolution operations by combining grouped convolution with pointwise spatial convolution. This design not only significantly reduces the model’s parameter count and computational overhead but also improves inference speed. Consequently, the model achieves efficient real-time detection in resource-constrained environments. Moreover, GSConv reduces the computational burden while largely preserving the original detection accuracy, demonstrating a favorable balance between efficiency and performance. Therefore, it is particularly well-suited for bird detection tasks that demand both computational efficiency and high accuracy.

**Figure 8:**
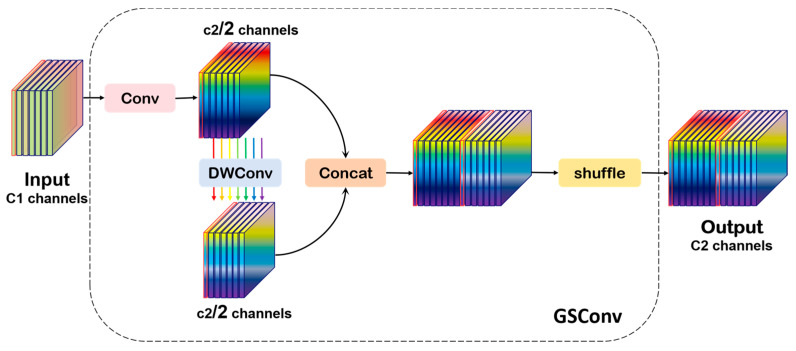
The structure of the GSConv module.

The GSConv module adopts a hybrid convolutional architecture that enables lightweight feature extraction by combining dense channel-wise connections with sparse spatial sampling. It maintains global channel interaction while employing sparse convolutions to aggregate contextual information and reduce computational redundancy. The module features a dual-branch structure: one branch uses standard convolution for downsampling and coarse-grained semantic capture, while the other applies depthwise convolution (DWConv) to extract fine-grained spatial details. The outputs are concatenated along the channel dimension and processed by a channel shuffle operation to promote cross-channel information exchange. This complementary fusion of global semantics and local textures enhances feature representation, as illustrated in Figure 8.

The feature extraction module employs a three-stage processing pipeline. First, a standard convolutional layer compresses the input feature map C1 by reducing its channel dimension to C2/2, which not only extracts primary feature representations but also decreases computational load for subsequent operations. Next, DWConv is applied independently across channels, leveraging a sparse sampling strategy to enhance feature diversity and improve the module’s capacity for fine-detail representation. Finally, the outputs from the standard convolution branch and the depthwise convolution branch are concatenated along the channel dimension, reconstructing the full C2 channel feature map. A subsequent channel shuffle operation rearranges the features to optimize their distribution, facilitating cross-channel interaction and fusion, thereby producing a more discriminative and robust feature representation. Its computational complexity formula is

(2) $$T_{GSC\mathrm{onv}}=W\times H\times (K_{1}^{2}\times C_{1}\times \frac{C_{2}}{2}+K_{2}^{2}\times \frac{C_{2}^{2}}{2}),$$

where K1, K2 is the convolution kernel size.

In practical bird detection scenarios at Dongting Lake, computing resources are often constrained, particularly in embedded systems or mobile devices with limited processing power and storage capacity. To address these limitations, the GSConv module is introduced, optimizing the convolutional structure to better utilize scarce computational resources and enable faster real-time inference. Not only boosts the overall detection performance but also improves the model’s applicability and practical value in resource-constrained environments.

## 3. Results and Analysis

### 3.1. Experimental Environment Configuration and Evaluation Indicators

Table 2 shows the experimental environment and parameter settings. All training conducted experiments and comparative analysis on the same dataset, experimental platform, and parameters. Due to limited computing resources and high training costs, all results are based on a fixed random seed of 0 to ensure reproducibility. To ensure fairness and consistency in the training outcomes, all ablation and comparative experiments were conducted without employing pre-trained weights.

In order to comprehensively evaluate and analyze the performance of the chili maturity detection model in terms of model compactness, accuracy, and real-time capability, several key metrics were selected for a multidimensional assessment. For model compactness, the evaluation primarily focused on specific indicators such as the number of parameters (Parameters), floating-point operations (FLOPs). In terms of model accuracy, the key evaluation metrics included precision (P), recall (R), and mean average precision (mAP). The mAP was computed by averaging the precision at an Intersection over Union (IoU) threshold of 0.5, denoted as mAP@0.5. The calculation formulas for these metrics are presented in Equations (3)–(6).

(3) $$P=\frac{TP}{TP+FP}\times 100\%,$$

(4) $$R=\frac{TP}{TP+FN}\times 100\%,$$

(5) $$mAP=\frac{\sum_{1}^{n}AP_{n}}{n}\times 100\%,$$

(6) $$F1\;\;S\mathrm{core}=\frac{2\times R\times P}{R+P},$$

### 3.2. Comparative Analysis of Improved Strategies

This paper proposes an improved network of Birds-YOLO for bird detection in Dongting Lake. Based on yolov11n architecture, the network improves the backbone and integrates the attention mechanism and RepNCSPELAN4-ECO module. In order to strictly evaluate the effectiveness of each improvement, we designed and carried out a series of comparative experiments to test the improvement strategies of different attention mechanisms and backbone networks.

#### 3.2.1. Comparison of Attention Mechanisms

We compared five different attention mechanisms—CBAM, CoT [37], SE [38], ELA [39], and CAA [40]—alongside the proposed EMA module. Each attention mechanism was integrated into the Birds-YOLO network, replacing the EMA module at the same position to ensure a fair comparison. The detailed experimental results are presented in Table 3.

The experimental results demonstrate that among all the compared attention mechanisms, the EMA achieves the best overall performance in the two key metrics of mAP and R. Although this precision is slightly lower than that achieved by networks using the SE and CoT attention mechanisms, it is important to note the different strengths of these mechanisms: the SE mechanism enhances accuracy by adaptively learning channel-wise weights to emphasize key channel information, while the CoT mechanism improves decision-making by mining context-related integrated information. Both contribute uniquely to improving accuracy. However, when considering the balance between model efficiency and accuracy comprehensively, EMA emerges as the most favorable choice under the current experimental conditions.

**Figure 9:**
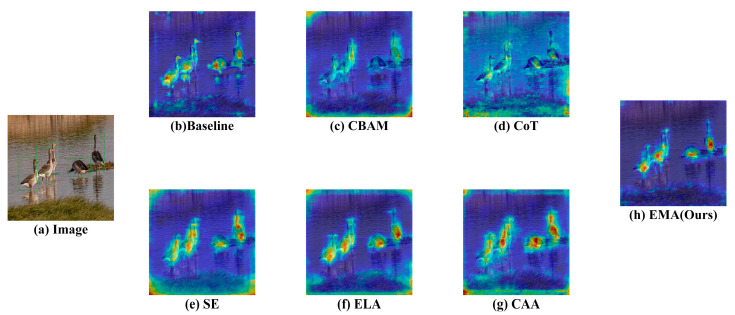
Visualization comparison of feature maps with different attention mechanisms. Sub-figure (**a**) is an image from the DTH-Birds test set; The subimage (**b**) shows the basic effect of the benchmark model; Subimages (**c**–**h**) display the heatmaps after adding CBAM, CoT, SE, ELA, CAA, and EMA attention modules, respectively.

In the thermal maps of different attention mechanisms as illustrated in Figure 9, the regions highlighted by EMA correspond more closely to the actual objects in the images, such as birds. Moreover, its attention distribution more precisely covers the key feature areas of the targets. This indicates that the EMA attention mechanism can more effectively capture critical target information while suppressing irrelevant background regions. Consequently, the model can allocate greater focus to task-relevant features during image processing, thereby enhancing its performance in bird detection.

#### 3.2.2. Comparison of Backbone Network Improvements

For the backbone network improvement strategy, four representative modules—C3k2, Context Guided, RepNCSPELAN4, and RepNCSPELAN4-ECO—were selected for comparative experiments to evaluate their performance and optimization potential across different scenarios. Systematic experiments were conducted on the DTH-Birds dataset, with the three strategies assessed and compared based on metrics including precision, recall, mAP, F1 score, Params, and FLOPs. The experimental results are summarized in Table 4.

The Context Guided backbone network performs better on the precision (P) metric because it emphasizes capturing and utilizing contextual information around the target during feature extraction. Through specific mechanisms such as the context guidance module, it can comprehensively integrate environmental information surrounding the target, enabling the model to make decisions based on richer and more complete context cues. This effectively reduces false positives, thereby improving precision.

From the perspective of model efficiency, the RepNCSPELAN4-ECO module exhibits significant advantages in terms of Params and FLOPs. The number of parameters for RepNCSPELAN4-ECO is 2.94 M, which is approximately 18.33% less than RepNCSPELAN4; Its FLOPs are about 29.03% lower than RepNCSPELAN4. This indicates that the RepNCSPELAN4-ECO module can significantly reduce the complexity and computational complexity of the model while maintaining a certain level of performance, which helps to reduce resource consumption during model training and inference processes, improve model deployment efficiency, and is particularly suitable for applications with limited computing resources.

In terms of performance indicators, although RepNCSPELAN4-ECO excels in P, R, mAP@0.5 and mAP@0.5:0.95, and other indicators were slightly lower than RepNCSPELAN4, but the difference was not significant. This small decrease in performance is usually acceptable in practical applications, especially when considering the significant efficiency improvement it brings.

### 3.3. Comparison Experiments

Birds-YOLO effectively detects multi-scale objects, including densely clustered targets, and demonstrates a superior ability to distinguish targets within complex backgrounds. This indicates that our model performs robustly when handling birds of varying sizes as well as those in densely populated and cluttered scenes. To further validate the effectiveness of Birds-YOLO, we compared its performance against other mainstream target detection algorithms. The experimental results on the CUB200-2011 dataset are shown in Table 5, while the results on the DTH-Birds dataset are presented in Table 6. From these results, it is evident that Birds-YOLO outperforms other mainstream YOLO series algorithms in terms of mAP. Specifically, Birds-YOLO achieved mAP@0.5 scores of 83.5% and 91.8% on the two datasets, respectively, ranking highest among all compared algorithms.

**Figure 10:**
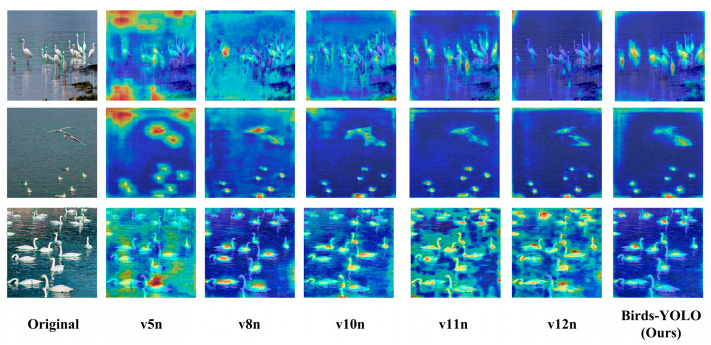
Visualization comparison of feature maps of different mainstream models. From left: original image and heatmaps from v5n, v8n, v10n, v11n, v12n, and our method. Birds-YOLO generates sharper and more accurate activations, focusing on bird bodies and reducing interference from complex backgrounds.

To more intuitively observe the capability of different models in capturing the features of bird targets during detection and processing, the feature heatmaps of models including YOLOv5n, YOLOv8n, YOLOv10n, YOLOv11n, and YOLOv12n are presented in Figure 10.

Through in-depth observation of these heatmaps, the advantages of the Birds-YOLO are clearly evident. In terms of target localization accuracy, the Birds-YOLO heatmaps align almost perfectly with the actual bird objects in the image, especially highlighting key feature regions. The thermal distribution closely matches the true shape and position of the targets.

**Figure 11:**
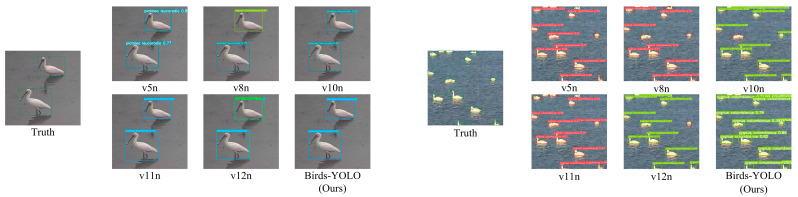
Model comparison diagram for false positive and missed detection problems. Green dashed box: indicates the real ground truth, that is, the birds that should be detected; Red dashed box: indicates false negatives, that is, the real target that the model cannot detect. (**Left**) Detection of Platalea leucorodia; V8n and v12n models have false detection, and the accuracy of the Birds-YOLO model is better than other models; (**Right**) Dense bird population on water surface; In view of the dense and complex background, some models have missed detection.

As shown in the left panel of Figure 11, we observe that several baseline models incorrectly classify Platalea leucorodia as other bird species, indicating poor discriminative power in fine-grained classification tasks. In contrast, Birds-YOLO can accurately identify and locate the target without any false alarms. Through enhanced feature representation and multi-scale feature fusion, it shows superior species recognition ability. At the same time, in the Dongting lake bird detection environment, there are a large number of birds and strong background clutter, and most of the existing models have some missed detection. However, Birds-YOLO achieves near perfect detection, highlighting its robustness to occlusion, scale change and visual interference. These results jointly verify that Birds-YOLO is superior to the most advanced detection model in terms of classification accuracy and detection accuracy.

In complex scenes, the Birds-YOLO demonstrates superior anti-interference capability. Even when the background contains challenging elements such as water and vegetation, the model effectively suppresses interference and maintains focused attention on the bird targets. The heat remains concentrated on the target areas, whereas other models tend to be distracted by background noise, resulting in scattered heatmaps or misdetections.

### 3.4. Ablation Experiments

To comprehensively and rigorously validate the effectiveness of the Birds-YOLO, we conducted detailed ablation experiments on the public CUB200-2011 dataset. In these experiments, key modules were progressively added according to the stepwise improvement of the model architecture, with the performance of the model recorded at each stage. The ablation results clearly demonstrate that as each carefully designed module is integrated, the key metric mAP of the model in the target detection task shows a significant and consistent improvement. This trend strongly indicates that the introduced modules play a positive and effective role in optimizing feature extraction and enhancing both target localization and classification capabilities. The detailed experimental results are presented in Table 7.

In the ablation experiments conducted on the private DTH-birds dataset, we followed the same experimental procedures and evaluation criteria as those used for the public dataset, ensuring the comparability and reliability of the results.

**Figure 12:**
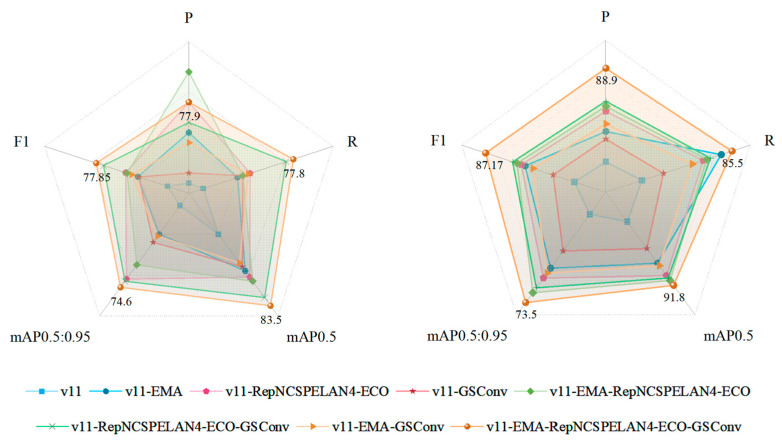
Radar image of Ablation Experiment on CUB200-2011 (**left**) and DTH-Birds (**right**).

As shown in Table 8, when all the proposed optimization modules were integrated, the Birds-YOLO achieved significant improvements in both mAP and F1 scores, reaching 91.8% and 87.17%, respectively. These results not only strongly validate the effectiveness of the Birds-YOLO in general detection scenarios but also demonstrate its robust adaptability and superior performance in real-world applications represented by specific private datasets. We present the results more intuitively through radar charts, as shown in Figure 12. This lays a solid foundation for its widespread use in practical ecological monitoring tasks.

The consistent improvement observed across all metrics suggests that the proposed modules are not only individually effective but also synergistic in nature.

## 4. Discussion

The Birds-YOLO model proposed in this paper integrates the EMA attention mechanism, an improved RepNCSPELAN4-ECO module, and lightweight GSConv convolution to address challenges in bird detection such as multi-scale targets, complex backgrounds, and efficiency. Experimental results show significant improvements in mAP, demonstrating the effectiveness of the approach on a self-built dataset from the Dongting Lake area. However, the added components increase computational complexity and model size, potentially limiting deployment in resource-constrained or real-time scenarios. Additionally, the dataset, while diverse, may not fully represent all bird species or environmental variations in the region, which could affect generalization. It should also be noted that, due to high training costs, experiments were conducted with a fixed random seed for reproducibility, limiting statistical analysis through multiple runs. Future work will focus on expanding datasets, exploring model compression techniques to reduce inference cost, and validating the framework’s generalizability to other ecological monitoring tasks such as detecting amphibians, insects, or small mammals under real-world conditions.

## 5. Conclusions

This study aims to propose a high-precision and robust bird detection model for the Dongting Lake area. We collected real-world data covering 47 species of birds in Dongting Lake through various methods and developed an improved bird detection model named Birds-YOLO based on this dataset.

First, an Efficient Multi-scale attention mechanism is introduced to address the insufficient feature extraction capacity of traditional backbone networks. This mechanism enhances semantic interaction across multi-scale feature maps via a dynamic weighting strategy. The EMA module adopts a parallel multi-branch architecture that integrates both channel and spatial attention, enabling efficient cross-scale feature fusion with low computational overhead. It effectively mitigates the performance bottleneck of traditional backbones in detecting small targets and complex backgrounds.

Second, to further improve feature extraction efficiency and multi-scale representation ability, we replaced the backbone network with a new architecture incorporating the RepNCSPELAN4-ECO module. This module not only enhances feature expression but also significantly improves multi-scale feature fusion efficiency, particularly in complex environments.

Finally, to reduce the computational redundancy in the neck structure, the GSConv module is introduced to replace and optimize traditional convolutions. GSConv combines grouped convolution with channel shuffling to significantly reduce computational complexity while maintaining accurate feature transmission. This improves the overall detection speed and resource utilization efficiency.

Experimental results show that Birds-YOLO outperforms the baseline model across multiple metrics. On the CUB200-2011 and DTH-Birds datasets, Birds-YOLO achieves mAP@0.5 scores of 83.5% and 91.8%, respectively, representing improvements of 3.5% and 2.6% over the original YOLOv11n. The proposed method demonstrates strong detection performance across diverse bird species, scales, and complex natural backgrounds, surpassing existing mainstream object detectors in overall effesctiveness.

## Figures and Tables

**Table 1:** Comparison table of two different data sets and data augmentation settings.

Dataset	Classes	Training Set	Validation Set	Test Set	Image Resolution
CUB-200-2011	200	8242	1773	1773	640 × 640
DTH-Birds	47	11,287	1410	1410	640 × 640
Data Augmentations					
Salt & Pepper Noise			2%		
Darker			×0.9		
Brighter			×1.5		
Random Rotation			±15°, ±30°		

**Table 2:** Experimental environment configuration.

Environment	Version
Operating system	Windows11
GPU	NVIDIA GeForce RTX 4080 SUPER
Python	3.8.2
Pytorch	2.3.1
CUDA	12.6
Epoch	300
Batch size	16
Optimizer	SGD
Initial learning rate	0.01
learning rate schedule	linear decay
Image size	640 × 640
Weight decay	0.0005
Momentum factor	0.937
Confidence threshold	0.5

**Table 3:** Performance results of different attention mechanisms on the DTH-Birds dataset.

Method	P/%	R/%	mAP@0.5/%	mAP@0.5:0.95/%	F1 Score/%	Params/M	FLOPs/G
v11n	77.1	72.8	80	70.6	74.89	2.58	6.3
v11n + CBAM	85.6	85.1	89.7	71.1	85.35	2.41	6.3
v11n + CoT	**87.1**	83.9	89.3	70	85.47	2.92	6.7
v11n + SE	86.7	84.9	90.1	71.4	85.79	2.35	6.3
v11n + ELA	83.7	84.4	89	70.8	84.05	2.41	6.3
v11n + CAA	86.2	84.1	90	70.9	85.14	2.48	6.4
v11n + EMA	86.4	**85.2**	**90.5**	**71.6**	**85.80**	**2.35**	**6.3**

Note: The best results are highlighted in **bold**.

**Table 4:** Performance results of different backbone networks on the DTH-Birds dataset.

Method	P/%	R/%	mAP@0.5/%	mAP@0.5:0.95/%	F1 Score/%	Params/M	FLOPs/G
C3k2	85.2	83.0	89.2	69.9	84.09	**2.58**	**6.3**
ContextGuided [41]	89.2	82.6	89.7	70.7	85.77	2.63	6.8
RepNCSPELAN4	**87.9**	**85.5**	**91.8**	**72.9**	**86.68**	3.60	12.4
RepNCSPELAN4-ECO	87.2	84.7	91.4	72.5	85.93	2.94	8.8

Note: the best results are highlighted in **bold**.

**Table 5:** Performance of different mainstream detection models on the CUB200-2011 dataset.

Method	P/%	R/%	mAP@0.5/%	mAP@0.5:0.95/%	F1 Score/%	Params/M	FLOPs/G
FasterRCNN	76.6	65.6	74.9	54.8	70.67	137.10	370.2
SSD	78.2	66.8	75.9	64.8	72.05	26.29	62.8
v5n	43.5	55.5	51.9	44.0	48.77	**1.82**	**4.3**
v8n	75.0	74.0	80.5	70.6	74.50	3.01	8.1
v10n	74.8	74.6	81.1	71.6	74.70	2.71	8.3
v11n	77.1	72.8	80.0	70.6	74.89	2.58	6.3
v12n	77.2	73.2	81.2	72.0	75.15	2.57	6.4
Ours	**77.9**	**77.8**	**83.5**	**74.6**	**77.85**	2.60	8.5

Note: The best results are highlighted in **bold**.

**Table 6:** Performance of different mainstream detection models on the DTH-Birds dataset.

Method	P/%	R/%	mAP@0.5/%	mAP@0.5:0.95/%	F1 Score/%	Params/M	FLOPs/G
FasterRCNN	81.3	75.8	84.6	66.2	78.45	137.1	370.2
SSD	82.1	76.3	86.2	67.4	79.09	26.29	62.8
v5n	84.3	82.3	88.2	69.1	83.29	**1.82**	**4.3**
v8n	87.5	84.7	90.4	70.5	86.08	3.01	8.1
v10n	87.3	83.5	90.7	71.2	85.36	2.71	8.3
v11n	85.2	83.0	89.2	69.9	84.09	2.58	6.3
v12n	87.5	77.0	88.4	70.1	81.91	2.57	6.4
Ours	**88.9**	**85.5**	**91.8**	**73.5**	**87.17**	2.60	8.5

Note: The best results are highlighted in **bold**.

**Table 7:** Performance of ablation experiments on the CUB200-2011 dataset.

Method	EMA	RepNCSPELAN4-ECO	GSConv	P/%	R/%	mAP@0.5/%	mAP@0.5:0.95/%	F1 Score/%	Params/M	FLOPs/G
v11n				77.1	72.8	80.0	70.6	74.89	2.58	6.3
v11n	√			77.6	74.7	81.8	72.0	76.12	2.35	6.3
v11n		√		77.9	75.4	82.1	74.2	76.63	2.94	8.8
v11n			√	77.2	75.0	81.6	72.4	76.08	2.51	6.4
v11n	√	√		**78.2**	75.0	82.3	73.5	76.57	2.68	8.6
v11n		√	√	77.7	77.4	83.1	74.3	77.55	2.84	8.7
v11n	√		√	77.5	75.3	81.4	72.1	76.38	**2.25**	**6.1**
v11n	√	√	√	77.9	**77.8**	**83.5**	**74.6**	**77.85**	2.60	8.5

Note: The best results are highlighted in **bold**. √ indicates that the corresponding module is included in the model.

**Table 8:** Performance of ablation experiments on the DTH-Birds dataset.

Method	EMA	RepNCSPELAN4-ECO	GSConv	P/%	R/%	mAP@0.5/%	mAP@0.5:0.95/%	F1 Score/%	Params/M	FLOPs/G
v11n				85.2	83.0	89.2	69.9	84.09	2.58	6.3
v11n	√			86.4	85.2	90.9	72.1	85.80	2.35	6.3
v11n		√		87.2	84.7	91.4	72.5	85.93	2.94	8.8
v11n			√	86.1	83.6	90.3	71.4	84.83	2.51	6.4
v11n	√	√		87.4	84.8	91.6	73.1	86.08	2.68	8.6
v11n		√	√	87.6	84.9	91.5	72.9	86.23	2.84	8.7
v11n	√		√	86.7	84.4	91.0	72.3	85.53	**2.25**	**6.1**
v11n	√	√	√	**88.9**	**85.5**	**91.8**	**73.5**	**87.17**	2.60	8.5

Note: The best results are highlighted in **bold**. √ indicates that the corresponding module is included in the model.

## Data Availability

DTH-birds dataset was approved by Hunan Natural Resources Affairs Center; DTH-birds dataset is available upon request, restricted by institutional privacy policies. CUB-200-2011 datasets can be obtained from their original papers.

## Abbreviations

The following abbreviations are used in this manuscript:

YOLO	You Only Look Once
Faster R-CNN	Faster Region-based Convolutional Neural Networks
RPN	Region Proposal Network
DETR	DEtection TRansformer
FPN	Feature Pyramid Network
MSCB	Multi-Scale Convolution Block
MSCAM	Multi-scale Channel Attention Module
ASPP	Atrous Spatial Pyramid Pooling
GNN	Graph Neural Network
MCA	Multidimensional Collaborative Attention
CBAM	Convolutional Block Attention Module
EMA	Efficient Multiscale Attention Mechanism
DWConv	Depthwise Separable Convolution
CUB200-2011	The Caltech-UCSD Birds-200-2011
SPPF	The Spatial Pyramid Pooling Fast
BCE	Binary Cross-Entropy
DFL	Distribution Focal Loss
CIoU	Complete Intersection over Union
P	Precision
R	Recall
mAP	Mean Average Precision
CoT	Contextual Transformer
SE	Squeeze-and-Excitation
ELA	Efficient Local Attention
CAA	Context Anchor Attention
